# Hematological Inflammatory Indices and the HALP Score for Pathogen Differentiation in Culture-Proven Late-Onset Neonatal Sepsis

**DOI:** 10.3390/children13040449

**Published:** 2026-03-25

**Authors:** Aydin Bozkaya, Asli Okbay Gunes, Hatice Busra Kutukcu Gul

**Affiliations:** 1 Department of Pediatrics, Faculty of Medicine, Harran University, Sanlıurfa 63290, Türkiye; 2 Department of Pediatrics, Division of Neonatology, Health Sciences University, Sancaktepe Şehit Prof. Dr. İlhan Varank Training and Research Hospital, Istanbul 34668, Türkiye; asliokbay@gmail.com; 3 Department of Pediatrics, Faculty of Medicine, Kahramanmaraş Sütçü İmam University, Kahramanmaraş 46040, Türkiye; busra_kutukcu93@hotmail.com

**Keywords:** sepsis, systemic inflammatory indices, HALP score

## Abstract

**Objective:** To evaluate the diagnostic and prognostic utility of the hemoglobin–albumin–lymphocyte–platelet (HALP) score and several systemic inflammatory indices derived from routine blood parameters—including the systemic immune-inflammation index (SII), platelet-to-lymphocyte ratio (PLR), pan-immune inflammation value (PIV), and systemic inflammatory response index (SIRI)—for pathogen differentiation and clinical assessment in culture-proven late-onset neonatal sepsis (LOS). **Methods:** A retrospective analysis was conducted on a cohort of 150 neonates with culture-proven LOS. Systemic inflammatory indices were calculated at baseline (first week of life) and at the time of septic insult. The discriminative power of these indices was assessed via ROC curve analysis, with optimal cut-off points determined by the Youden Index. Risk stratification was performed using Odds Ratio (OR) modeling with 95% Confidence Intervals (CIs) to evaluate the predictive strength of each marker according to its respective threshold. **Results:** Diagnosis-phase assessments identified SII as the premier discriminator for microbiological etiology (AUC = 0.869; OR = 44.57), outperforming PLR and PIV. Although HALP demonstrated moderate efficacy in distinguishing pathogens, it lacked prognostic value regarding mortality. Conversely, SIRI displayed limited clinical utility, yielding the lowest predictive performance in our cohort. **Conclusions:** In neonatal sepsis, the HALP score provided additional clinical information when compared with several hematological inflammatory indices. Although HALP was not associated with mortality, prospective multicenter studies are needed to clarify the role of these cost-effective markers in pathogen differentiation and clinical assessment of LOS.

## 1. Introduction

Neonatal sepsis continues to be a formidable driver of morbidity and mortality, posing a disproportionate risk to preterm and low-birth-weight infants hospitalized in neonatal intensive care units (NICUs) [1]. The condition is typically categorized based on the timing of onset: early-onset sepsis, which presents within the first 72 h of life, and late-onset sepsis (LOS), which manifests after this initial 72 h window. Unlike early-onset cases, LOS is predominantly linked to hospital-acquired pathogens and environmental exposures within the NICU, necessitating vigilant monitoring and rapid diagnostic intervention. LOS is characterized by a more complex clinical course in preterm and very low birth weight neonates, largely due to prolonged hospitalization, invasive procedures, and immature immune function [1,2,3].

The pathophysiology of sepsis is multifactorial, involving systemic inflammation alongside hematological, immunological, and nutritional alterations [1]. Although blood culture remains the diagnostic gold standard, delays in results, false-negative findings, and the frequent occurrence of culture-negative sepsis have increased reliance on laboratory biomarkers for early and accurate diagnosis [4]. In addition to traditional markers such as C-reactive protein (CRP) and procalcitonin (PCT), hematological inflammatory indices derived from routine blood parameters, such as the neutrophil-to-lymphocyte ratio (NLR) and platelet-to-lymphocyte ratio (PLR), have gained increasing attention due to their rapid availability, low cost, and potential diagnostic and prognostic value in neonatal sepsis [5,6]. Among these, the PLR has demonstrated promising predictive value for sepsis diagnosis and severity assessment [7,8]. The hemoglobin–albumin–lymphocyte–platelet (HALP) score, extensively studied as a prognostic marker in malignancies, reflects nutritional status and immune hematological response but remains underexplored in neonatal sepsis [9,10,11]. Given the immaturity of the neonatal immune system, inflammatory responses differ substantially from those in older populations, necessitating age-specific validation of biomarkers. Whether HALP and systemic inflammatory indices are associated with pathogen characteristics or prognosis in culture-positive late-onset neonatal sepsis remains unclear, with limited data available in the literature [12].

Systemic inflammation plays an important role in the prognosis of many diseases, and several hematological indices derived from complete blood count parameters have been proposed to reflect the balance between inflammatory and immune responses, including SII, PLR, PIV, and SIRI. The HALP score, calculated from hemoglobin, albumin, lymphocyte, and platelet levels, differs from these indices by incorporating markers of both inflammation and nutritional status. Although HALP has been widely investigated in oncological and surgical settings, its role in neonatal sepsis remains poorly understood. Therefore, evaluating the relationship between HALP and established inflammatory indices such as SII, PLR, PIV, and SIRI may provide additional insight into its potential clinical relevance in neonatal sepsis [9,10,11,12].

The objective of this study was to investigate the diagnostic performance and prognostic value of the hemoglobin–albumin–lymphocyte–platelet (HALP) score alongside novel systemic inflammatory markers—specifically the systemic immune-inflammation index (SII), platelet-to-lymphocyte ratio (PLR), pan-immune inflammation value (PIV), and systemic inflammatory response index (SIRI). We focused on their capacity to discriminate between Gram-positive and Gram-negative etiologies in neonates with culture-proven late-onset sepsis (LOS). By establishing evidence-based cutoff values, this research seeks to refine early clinical decision-making, providing a framework for risk stratification and the optimization of empirical antimicrobial therapy during the critical interval preceding culture confirmation.

## 2. Materials and Methods

### 2.1. Study Design and Population

A retrospective analysis was performed on a descriptive cohort of neonates admitted to an NICU at a specialized training and research center between January 2020 and January 2024. The study utilized a multi-source data collection approach, integrating infection control registries with digitized medical records and bedside clinical notes to ensure data tripartite validation.

### 2.2. Inclusion and Exclusion Criteria

The study cohort strictly comprised neonates with microbiologically confirmed Late-Onset Sepsis (LOS). To ensure diagnostic specificity, cases involving culture-negative presentations, non-bacterial isolates, or growth restricted to extra-hematogenous specimens were systematically excluded. Furthermore, clinical sepsis cases lacking corroborative laboratory markers were omitted to maintain the integrity of the culture-proven dataset.

### 2.3. Data Collection

Baseline demographic characteristics encompassed neonatal sex, gestational age, and birth weight, alongside maternal age and the specific mode of delivery. Clinical parameters evaluated across the cohort included the duration of hospitalization and ultimate discharge outcomes. Furthermore, detailed clinical profiles were constructed by assessing the modality and duration of respiratory support, the administration of inotropes, associated risk factors, and the precise chronological onset of sepsis. Laboratory values were obtained from standardized routine blood tests performed at two predefined time points: during the first week of hospitalization (days 1–7) and at the time of sepsis diagnosis. Routine laboratory parameters included hemoglobin, albumin, platelet count, mean platelet volume, lymphocyte count, absolute neutrophil count, monocyte count, eosinophil count, basophil count, and total white blood cell count. Additional biochemical and inflammatory markers included creatinine, blood urea nitrogen, aspartate aminotransferase, alanine aminotransferase, alkaline phosphatase, gamma-glutamyl transferase, CRP, and PCT. Blood cultures were obtained at the time of suspected sepsis according to standard microbiological procedures.

### 2.4. Outcomes and Microbiological Classification

Clinical prognosis was determined by mortality rates and the necessity of cardiorespiratory interventions (inotropic and oxygen support). Following a comprehensive review of positive blood culture registries, isolated pathogens were stratified into Gram-positive and Gram-negative cohorts. Within the Gram-negative group, a more granular analysis was performed to distinguish between fermentative and non-fermentative bacterial strains.

### 2.5. Inflammatory Indices

To investigate the predictive capacity of systemic inflammatory indices in pathogen differentiation and neonatal prognosis, several composite biomarkers were derived from complete blood count (CBC) parameters. The indices assessed were as follows: HALP score = (hemoglobin × albumin × lymphocyte count)/platelet count; platelet-to-lymphocyte ratio (PLR); systemic immune-inflammation index (SII) = platelets × neutrophils/lymphocytes; pan-immune-inflammation value (PIV) = platelets × neutrophils × monocytes/lymphocytes; systemic inflammatory response index (SIRI) = neutrophils × monocytes/lymphocytes; neutrophil-to-lymphocyte ratio (NLR); monocyte-to-lymphocyte ratio (MLR).

### 2.6. Ethical Approval

The study protocol received formal approval from the Local Ethics Committee of Harran University (Number: 12; Date: 30 June 2025). All clinical procedures and data collection methods were performed in compliance with the principles of the Declaration of Helsinki regarding human subject research.

### 2.7. Statistical Analysis

All statistical analyses were performed using IBM SPSS Statistics for Windows, version 26.0 (IBM Corp., Armonk, NY, USA). The threshold for statistical significance was predefined as a two-tailed *p*-value < 0.05. Data distribution was rigorously assessed using the Shapiro–Wilk test; since the continuous variables exhibited non-normal distributions, they were expressed as medians with interquartile ranges (IQR, Q1–Q3), and non-parametric analytical frameworks were adopted. For categorical data, frequencies and percentages were compared using the Chi-square test, incorporating Yates’ correction or Fisher’s exact test where necessitated by cell counts. Intersubject differences for continuous metrics were evaluated using the Mann–Whitney U test for binary comparisons and the Kruskal–Wallis test for multi-group analyses, followed by Bonferroni-adjusted post hoc pairwise testing where global significance was achieved. The diagnostic utility of inflammatory indices in differentiating Gram-positive from Gram-negative etiologies was determined through Receiver Operating Characteristic (ROC) curve analysis, with optimal thresholds identified via the Youden Index. ROC analysis requires defining one group as a reference; therefore, Gram-negative infections were used as the reference category, while the analysis was based on comparisons between both groups. Subsequently, binary variables derived from these cutoffs were compared using Chi-square analysis to calculate Odds Ratios (OR) and 95% Confidence Intervals (CI). Notably, multivariate modeling was intentionally omitted to prevent statistical distortion arising from multicollinearity, as the calculated indices utilize overlapping primary variables.

## 3. Results

### 3.1. Demographic, Clinical and Laboratory Findings

**Figure 1:**
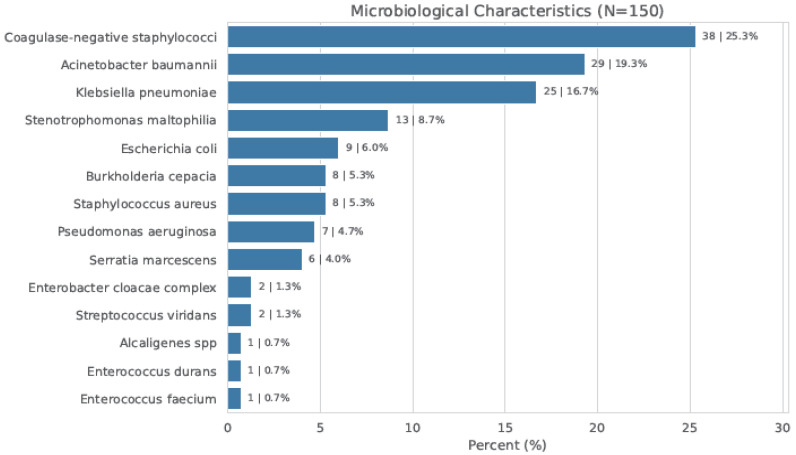
Microbiological characteristics.

Out of 6098 neonatal admissions screened over the four-year study interval, 525 (8.6%) developed systemic sepsis. Following strict enrollment criteria, 150 cases (2.4%) of definitive bacterial LOS were analyzed. Regarding the etiology, Gram-negative bacteria were twofold more prevalent than Gram-positive isolates (66.7% vs. 33.3%). Notably, within the Gram-negative group, fermentative and non-fermentative species showed an identical distribution. The most frequent isolates complicating the clinical course were coagulase-negative staphylococci, *Acinetobacter* spp., and *Klebsiella pneumoniae*, representing 25.3%, 19.3%, and 16.7% of the cohort, respectively (Figure 1).

The demographic analysis revealed that nearly half of the neonates were male (46%), and the majority were of Turkish origin (85.3%). High rates of cesarean delivery (79.3%) were observed within the cohort. Neonatal maturity and growth parameters showed a mean gestational age of 31 ± 5 weeks and a mean birth weight of 1648 ± 944, reflecting a predominantly preterm and low-birth-weight study group. Maternal age spanned from 16 to 49 years, with a mean of 29 ± 7. Notably, the manifestation of Late-Onset Sepsis (LOS) occurred at a median of 20 days post-birth (26 ± 21 days), extending up to 130 days in some instances.

Both study groups exhibited comparable demographic profiles, with no significant differences identified in sex, ethnic background, or maternal and neonatal anthropometric measurements. Although the frequency of cesarean sections was higher among neonates with Gram-positive infections (88% vs. 75%), the discrepancy was statistically negligible (*p* = 0.101). A marginal trend toward delayed sepsis onset was noted in the Gram-positive group, yet this did not achieve the threshold for significance (*p* = 0.078). In terms of clinical interventions, the necessity for oxygen therapy and inotropic agents showed no significant deviation between the two cohorts (Table 1).

A significant discrepancy was observed in the frequency of central venous catheterization, which was notably higher among neonates with Gram-positive infections than those with Gram-negative etiologies (76% vs. 51%; *p* = 0.006). No other significant differences were identified across the remaining risk factor profiles (all *p* > 0.05), indicating a homogeneous distribution of baseline clinical risks between the two microbiological subsets.

Clinical symptoms such as vomiting, gastric residual volume, and apnea were more prevalent among infants with Gram-positive infections, yet these trends remained statistically non-significant (*p* > 0.05). Notably, the incidence of necrotizing enterocolitis was markedly elevated in the Gram-positive cohort at 20%, compared to only 3% in the Gram-negative cohort (*p* = 0.001). Analysis of other clinical comorbidities revealed no additional statistically significant variances between the two groups, as detailed in Table 1.

Of the 150 neonates enrolled, 45 (30%) succumbed to the infection, whereas 96 (64%) reached discharge status. A comparative analysis of the microbiological subgroups revealed a high degree of equivalence in survival outcomes. The incidence of mortality was statistically identical between those with Gram-positive and Gram-negative pathogens, yielding a *p*-value of 1.000 (Table 1).

Hematological assessments at clinical presentation showed that Gram-positive sepsis was associated with significantly higher monocyte levels (*p* = 0.017) and increased ANCs (*p* = 0.020). In contrast, Gram-negative sepsis was characterized by a more severe hematological insult, evidenced by significantly lower platelet counts (*p* < 0.001 and a more robust inflammatory response. This was further corroborated by CRP and PCT levels, both of which were significantly higher in the Gram-negative subset compared to the Gram-positive cohort (*p* < 0.001 and *p* = 0.012, respectively; Table 2).

While pre-sepsis albumin concentrations were comparable across both cohorts (*p* = 0.675), a significant decline was observed in the Gram-negative group at the time of clinical diagnosis (*p* = 0.003). Regarding hepatic markers, AST levels were significantly more elevated in neonates with Gram-negative sepsis at the onset of infection (*p* = 0.017). Conversely, ALT levels remained statistically similar between the two microbiological subsets at diagnosis, showing no significant intergroup disparity (*p* = 0.128) (Table 2).

Inflammatory indices calculated at the time of diagnosis showed generalized elevations across the study population. Specifically, NLR-2 values demonstrated no significant difference between the microbiological subsets (*p* = 0.319). This lack of statistical divergence was also mirrored in the MLR-2 values (*p* = 0.292). These findings indicate that both NLR and MLR respond robustly to the septic insult, regardless of whether the underlying pathogen is Gram-positive or Gram-negative (Table 2).

A distinct divergence was observed in the more complex systemic inflammatory markers. Specifically, PLR-2, SII-2, and PIV-2 levels were significantly more elevated in neonates with Gram-positive infections (all *p* < 0.001). In stark contrast, SIRI-2 and particularly HALP-2 values exhibited a significant predominance in the Gram-negative cohort (*p* = 0.006 and *p* < 0.001, respectively). These findings suggest that while certain indices align with Gram-positive responses, others—most notably the HALP-2 score—may serve as more sensitive indicators for Gram-negative bacterial insults.

The longitudinal assessment of the HALP index revealed that while pre-infectious levels (HALP-1) were statistically equivalent across groups (*p* = 0.962). diagnosis-phase values (HALP-2) exhibited a significant microbiological dependency. Specifically, neonates with Gram-positive sepsis presented with markedly lower HALP-2 scores than those with Gram-negative etiologies (4.7 vs. 14.9; *p* < 0.001) This nearly threefold difference in HALP-2 values underscores the index’s potential utility in early pathogen differentiation within the NICU setting (Table 2).

The impact of bacterial etiology on HALP scores was further scrutinized within the exitus cohort. Median HALP-1 levels demonstrated remarkable consistency across Gram-negative fermentative (8.6), Gram-negative non-fermentative (9.2), and Gram-positive (8.7) groups (all *p* > 0.05). Evaluation of HALP-2 values—ranging from a median of 4.6 in Gram-positives to 31.0 in non-fermentative Gram-negatives—likewise failed to reach statistical significance (*p* > 0.05). Consequently, neither the baseline nor the diagnostic-phase HALP index appeared to vary significantly according to the underlying pathogen in fatal cases, suggesting a uniform systemic insult across microbiological etiologies (Table 2).

### 3.2. Analyses Between the Scorings

**Figure 2:**
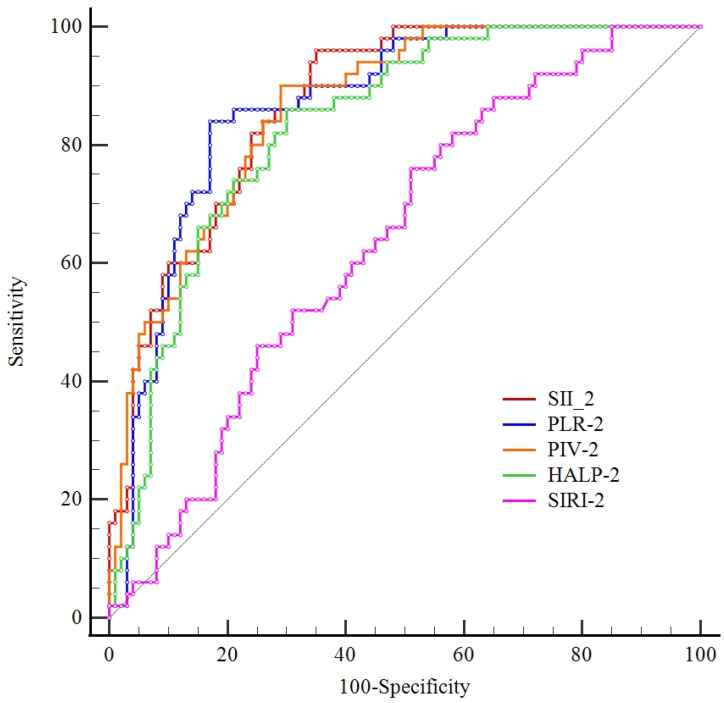
ROC curve analysis of the discriminatory power of inflammatory biomarkers.

The diagnostic performance of inflammatory indices in identifying Gram-positive infections was evaluated via ROC curve analysis. Both SII-2 (AUC: 0.869) and PLR-2 (AUC: 0.866) demonstrated superior discriminative capacity. Specifically, an SII-2 threshold of >120 yielded a remarkably high sensitivity of 96%, while a PLR-2 cutoff of >44 provided a balanced diagnostic profile with 84% sensitivity and 83% specificity. PIV-2 (AUC: 0.863) and HALP-2 (AUC: 0.832) also exhibited robust accuracy, with the latter achieving 86% sensitivity at a cutoff of ≤8.64. In contrast, SIRI-2 demonstrated a relatively limited predictive value, characterized by a lower AUC of 0.637 and a specificity of only 49% (Figure 2, Table 3).

HALP = (Hemoglobin × Albumin × Lymphocyte count)/Platelet count, PLR (Platelet-to-Lymphocyte Ratio), SII (Systemic Immune-Inflammation Index) = Platelets × Neutrophils/Lymphocytes, PIV (Pan-Immune-Inflammation Value) = Platelets × Neutrophils × Monocytes/Lymphocytes, SIRI (Systemic Inflammation Response Index) = Neutrophils × Monocytes/Lymphocytes.

Risk stratification analysis revealed that elevated systemic indices were potent predictors of Gram-positive etiology. Most notably, an SII-2 value exceeding 120 was associated with a staggering 44.57-fold increase in the likelihood of Gram-positive infection (OR: 44.57; 95% CI: 10.22–194.43). Strong predictive associations were also observed for PLR-2 > 44 and PIV-2 > 190, which increased the risk by 23.92-fold (OR: 23.92; 95% CI: 9.61–59.54) and 22.00-fold (OR: 22.00; 95% CI: 7.95–61.10), respectively. Furthermore, a HALP-2 score of ≤9 conferred a 14.33-fold higher risk (OR: 14.33; 95% CI: 5.79–35.47); although this effect size was more modest than that of the other multi-lineage indices, it remained both statistically robust and clinically significant. In contrast, SIRI-2 > 1.65 demonstrated a comparatively weaker association, with only a 3.00-fold increase in risk (OR: 3.00; 95% CI: 1.43–6.49) (Table 4).

## 4. Discussion

Despite substantial refinements in neonatal intensive care, sepsis continues to be a formidable driver of morbidity and mortality, necessitating the identification of rapid, bedside biomarkers for early severity stratification. This study represents a novel attempt to integrate the HALP score with emerging systemic inflammatory indices—such as SII, PIV, and SIRI—to evaluate their combined utility in predicting prognosis and differentiating microbiological etiologies in neonatal Late-Onset Sepsis (LOS). The HALP score integrates nutritional status and immune hematological response and has been proposed as a prognostic marker in oncological and cardiovascular populations [9,10,11]. In the present study, HALP showed moderate discriminatory ability for pathogen differentiation but was not independently associated with mortality. While lower HALP scores have been linked to increased mortality in adult sepsis [13,14], this association was not observed in our neonatal cohort, possibly due to neonatal-specific factors such as immune immaturity, comorbidities, and intensive supportive therapies. Conflicting findings regarding HALP behavior in Gram-negative infections further emphasize the need for age-specific validation [15]. Owing to its cost-effectiveness and reliance on routinely available hematological parameters, the HALP score holds significant promise as a complementary bedside tool. Especially during the hyperacute phase of sepsis, it may facilitate early risk stratification and guide empirical antimicrobial stewardship in conjunction with established neonatal scoring systems. However, while HALP provides valuable surrogate data, definitive clinical evaluation and microbiological confirmation must remain the cornerstones of neonatal sepsis management.

A study reported that NLR and PLR values showed a significant positive correlation with defined sepsis prognostic scores [6]. However, the role of these indices in distinguishing pathogen types has not yet been clearly demonstrated. In our study, significant differences were observed in inflammation indices at the onset of sepsis. Although the NLR at diagnosis was high in both groups and did not show a significant difference, the significantly higher PLR value in Gram-positive bacteria is important information regarding its potential use in pathogen differentiation.

Among the evaluated markers, SII demonstrated the highest diagnostic accuracy and emerged as a strong discriminator of causative pathogens. In contrast, NLR and MLR did not differ significantly between Gram-positive and Gram-negative infections, suggesting limited value for pathogen-specific differentiation despite reflecting overall inflammatory burden [16,17]. PLR, a well-established inflammatory marker in sepsis and other systemic diseases [18], also showed strong discriminatory performance in LOS. Among newer composite indices, PIV demonstrated substantial diagnostic utility, whereas SIRI exhibited weaker performance, indicating limited applicability in this clinical context [19].

Similarly, previous studies have reported that systemic inflammatory indices such as NLR, PLR, MLR, SII, SIRI, and PIV are associated with disease severity and adverse clinical outcomes in neonatal critical conditions [6]. In a recent study involving neonates with hypoxic–ischemic encephalopathy, these indices were also shown to correlate with disease severity, supporting the clinical relevance of hematological inflammatory markers in neonatal disorders [20]. These composite indices integrate leukocyte and platelet parameters and therefore reflect the balance between systemic inflammation and immune response. During neonatal sepsis, activation of innate immunity leads to neutrophilia and platelet activation, whereas lymphopenia may occur due to immune dysregulation and stress response. Consequently, indices combining these parameters may better capture the overall inflammatory burden than single laboratory markers [7]. Although SII demonstrated strong discriminatory performance in our cohort, the HALP score may provide additional clinical insight by incorporating hemoglobin and albumin levels alongside lymphocyte and platelet counts, thereby reflecting both inflammatory and nutritional status. Consistent with these observations, our findings suggest that the HALP score may offer complementary information among inflammation-based indices and may be particularly useful for pathogen differentiation and prognostic assessment in neonatal sepsis.

Neonatal hematological parameters change rapidly in the early postnatal period and can be influenced by perinatal factors such as gestational age and intrauterine conditions. For this reason, first-week values in our study were used as an individual baseline rather than a true physiological control, allowing a more reliable evaluation of sepsis-related hematological changes within the same patient [21]. Although CRP and PCT remain essential for sepsis diagnosis and monitoring [6,22], their ability to distinguish pathogen type is limited. In this study, both markers were higher in Gram-negative infections, consistent with endotoxin-driven inflammation [23], supporting the use of composite inflammatory indices alongside conventional biomarkers while awaiting culture results.

The primary strength of this investigation lies in its stringent inclusion criteria, focusing exclusively on culture-proven LOS cases to ensure microbiological accuracy. Furthermore, this study provides a uniquely comprehensive comparative analysis of multiple systemic inflammatory indices alongside the HALP score, offering a multidimensional perspective on pathogen differentiation and prognosis. However, several limitations must be acknowledged, including its retrospective, single-center design and the relatively modest sample size. Additionally, the potential confounding influence of extreme prematurity, varying nutritional statuses, and diverse supportive interventions on individual HALP components warrants cautious interpretation of these markers in heterogeneous neonatal populations.

## 5. Conclusions

In conclusion, this study underscores the significant potential of hematological inflammatory indices as cost-effective, readily accessible tools for early pathogen differentiation and risk stratification in neonatal Late-Onset Sepsis. Among the parameters evaluated, the Systemic Immune-Inflammation Index (SII) demonstrated superior discriminative power, followed by PLR and PIV. While the HALP score offered moderate diagnostic utility, its unique value lies in reflecting the complex interplay between nutritional status and immune response. When integrated with clinical judgment, these indices can refine empirical antimicrobial strategies and optimize resource allocation in the NICU while awaiting definitive microbiological results. Nevertheless, further prospective, multicenter trials are essential to standardize these markers and validate their integration into routine neonatal clinical practice.

## Figures and Tables

**Table 1:** Demographic, clinical, and treatment characteristics of late neonatal sepsis cases.

Characteristic	Total n = 150 N (%)/Med (IQR)	Gram (+) n = 50 n (%)/Med (IQR)	Gram (-) n = 100 n (%)/Med (IQR)	*p*-Value
Gender (male)	69 (46%)	24 (48%)	45 (45%)	0.862
Nationality				
Turkish	128 (85%)	46 (92%)	82 (82%)	0.165
Other	22 (15%)	4 (8%)	18 (18%)	
Delivery Mode (Cesarean section)	119 (79%)	44 (88%)	75 (75%)	0.101
Maternal age (years)	29 (24–35)	29 (25–35)	29 (24–35)	0.572
Gestational age (weeks)	30 (27–36)	31 (27–36)	30 (27–36)	0.667
Birth weight (g)	1220 (900–2400)	1100 (925–2000)	1315 (865–2550)	0.419
Sepsis onset days	20 (10–35)	25 (13–36)	18 (10–35)	0.078
**Risk factors**				
Umbilical catheter	90 (60%)	31 (62%)	59 (59%)	0.860
Central Catheter	89 (59%)	38 (76%)	51 (51%)	0.006
Abdominal drain	10 (7%)	5 (10%)	5 (5%)	0.302
Extraventricular drain	6 (4%)	2 (4%)	4 (4%)	1.000
Chest tube	3 (2%)	0 (0%)	3 (3%)	0.551
**Symptoms**				
Vomiting	43 (29%)	18 (36%)	25 (25%)	0.225
Residual	37 (25%)	13 (26%)	24 (24%)	0.947
Fever	38 (25.3%)	11 (22%)	27 (27%)	0.642
Apnea	24 (16%)	10 (20%)	14 (14%)	0.479
Hypotension	16 (10.7%)	3 (6%)	13 (13%)	0.304
Bradycardia	3 (2%)	2 (4%)	1 (1%)	0.258
**Treatment Characteristics**				
Oxygen support	148 (98.7%)	50 (100%)	98 (98%)	0.553
Inotropic support	115 (76.7%)	35 (70%)	80 (80%)	0.246
**Comorbidities**				
Prematurity	87 (58%)	31 (62%)	56 (56%)	0.599
Respiratory distress	82 (55%)	30 (60%)	52 (52%)	0.451
Bronchopulmonary dysplasia	30 (20%)	13 (26%)	17 (17%)	0.279
Congenital heart disease	24 (16%)	11 (22%)	13 (13%)	0.238
Intracranial hemorrhage	14 (9%)	6 (12%)	8 (8%)	0.552
Pulmonary hemorrhage	14 (9%)	5 (10%)	9 (9%)	1.000
Necrotizing enterocolitis	13 (9%)	10 (20%)	3 (3%)	0.001
Hydrocephalus	11 (7%)	3 (6%)	8 (8%)	0.752
Asphyxia	7 (5%)	3 (6%)	4 (4%)	0.686
Acute renal failure	6 (4%)	4 (8%)	2 (2%)	0.096
**Survival** (Exitus)	45 (30.0%)	15 (30.0%)	30 (30.0%)	1.000

Med: Median, IQR: Interquartile Range.

**Table 2:** Laboratory values and inflammatory markers by groups.

		Gram (+) (n = 50) Med. (IQR)	Gram (-) (n = 100) Med. (IQR)	*p*-Value
**Hematological Parameters**				
WBC (/μL)	1	11,495 (7410–15,680)	10,475 (7080–15,065)	0.400
	2	12,715 (8220–17,290)	9825 (5025–15,915)	0.037
Lymphocyte (/μL)	1	4355 (2510–5410)	3955 (2915–5390)	0.906
	2	3180 (1950–4320)	2465 (1490–4315)	0.126
Eosinophil (/μL)	1	230 (150–401)	210 (130–335)	0.481
	2	220 (115–360)	205 (110–333)	0.031
Basophil (/μL)	1	80 (40–120)	75 (40–120)	0.889
	2	60 (30–100)	60 (30–95)	0.351
MPV (fl)	1	10.5 (9.6–10.6)	10.1 (9.5–10.5)	0.108
	2	11 (10.4–11.8)	11 (10.5–12)	0.655
Monocyte (/μL)	1	1200 (740–1870)	1215 (730–1780)	0.959
	2	1645 (1120–2400)	1215 (740–1945)	0.017
ANC (/μL)	1	4795 (2390–8620)	3985 (1875–7080)	0.154
	2	5725 (3800–9360)	4345 (1825–7985)	0.020
Hemoglobin (g/dL)	1	15.7 (13.6–17.8)	15.9 (14.2–18.0)	0.489
	2	11.4 (10.4–12.7)	11.4 (10.1–13.3)	0.759
Platelet (/μL)	1	222,000 (176,000–261,000)	216,500 (177,000–284,500)	0.723
	2	195,000 (159,000–267,000)	50,500 (12,500–85,500)	<0.001
**Biochemical Markers**				
CRP (mg/L)	1	1.9 (0.5–4.0)	0.8 (0.5–3.0)	0.124
	2	55.0 (25.0–80.0)	90.0 (49.0–144.5)	<0.001
PCT (mg/L)	1	2.0 (0.9–4.0)	1.5 (0.5–5.7)	0.511
	2	15.0 (9.0–24.0)	22.4 (10.0–51.0)	0.012
Albumin (g/L)	1	31 (28–34)	30 (28–33)	0.675
	2	27 (24–31)	25 (21–28)	0.003
**Kidney and Liver Function Tests**				
BUN (mg/dL)	1	38.0 (26.1–54.0)	40.0 (27.0–49.5)	0.797
	2	44 (32–84)	52 (31–81)	0.404
Creatinine (mg/dL)	1	0.7 (0.5–0.8)	0.6 (0.5–0.9)	0.920
	2	0.6 (0.4–0.9)	0.7 (0.4–1.2)	0.570
ALP (U/L)	1	176 (150–210)	177 (151–209)	0.909
	2	220 (176–272)	210 (175–256)	0.070
GGT (U/L)	1	98 (65–140)	99 (72–144)	0.333
	2	108 (60–140)	102 (65–132)	0.409
Total Protein (g/L)	1	39 (36–43)	39.5 (36–45)	0.733
	2	39 (36–45)	39 (35–42)	0.162
AST (U/L)	1	52 (34–82)	48 (30–69)	0.463
	2	36 (24–83)	56 (38–86)	0.017
ALT (U/L)	1	16 (12–22)	11 (7–20)	0.020
	2	17 (11–39)	25 (15–44)	0.128
**Inflammatory Indices**				
PLR	1	60 (45–81)	60 (40–88)	0.919
	2	75 (48–106)	20 (7–35)	<0.001
SII	1	248 (138–547)	213 (114–416)	0.378
	2	507 (194–764)	70 (21–169)	<0.001
NLR	1	1.20 (0.63–2.39)	0.99 (0.48–1.64)	0.180
	2	2.01 (1.13–3.13)	1.74 (0.87–3.35)	0.319
MLR	1	0.26 (0.19–0.59)	0.32 (0.20–0.46)	0.848
	2	0.54 (0.30–0.76)	0.45 (0.30–0.74)	0.292
PIV	1	351 (106–794)	271 (99–609)	0.411
	2	660 (262–1619)	75 (21–234)	<0.001
HALP	1	7.9 (5.9–10.8)	7.8 (5.7–11.3)	0.962
	2	4.7 (2.9–6.7)	14.9 (6.7–40.8)	<0.001

Med: Median, IQR: Interquartile Range. Abbreviations: hemoglobin (Hg), albumin (Alb), lymphocyte (L), platelet (PLT), absolute neutrophil count (ANC), white blood cell (WBC), MPV (mean platelet volume), PCT, c-reactive protein (CRP), blood urea nitrogen (BUN), creatinine, aspartate aminotransferase (AST) and alanine aminotransferase (ALT), gamma glutamyl transferase (GGT), alkaline phosphatase (ALP), SIRI (Systemic Inflammatory Response Index), PLR (Platelet/Lymphocyte Ratio), PIV (Pan-Inflammatory Index), HALP (Hemoglobin, Albumin, Lymphocyte, Platelet Index), SII (Systemic Immune-Inflammation Index), NLR (neutrophil-to-lymphocyte ratio), and MLR (monocyte-to-lymphocyte ratio).

**Table 3:** ROC curve analysis of inflammatory biomarkers for discriminating between Gram-positive and gram-negative infections.

Variable	AUC (95% CI)	*p*-Value (Area = 0.5)	Youden Index (J)	Cut-Off Value	Sensitivity (%)	Specificity (%)
PLR-2	0.866 (0.801–0.916)	<0.001	0.67	>44	84	83
SII_2	0.869 (0.805–0.919)	<0.001	0.61	>120	96	65
SIRI-2	0.637 (0.554–0.714)	0.003	0.25	>1.65	76	49
PIV-2	0.863 (0.798–0.914)	<0.001	0.61	>189	90	71
HALP-2	0.832 (0.763–0.888)	<0.001	0.56	≤8.64	86	70

ROC: Receiver Operating Characteristic, AUC: Area Under the (ROC) Curve, SIRI-2 (systemic inflammatory response index), PLR-2 (Platelet/lymphocyte ratio), PIV-2 (pan-inflammatory index), HALP-2 (Hemoglobin, Albumin, Lymphocyte, Platelet index), and SII-2 (Systemic immune-inflammation index).

**Table 4:** Associations between inflammatory biomarkers and bacterial infections.

Variable	Cut-Off	Gram (+) n (%)	Gram (-) n (%)	*p*-Value	Odds Ratio (95% CI)	MH *p*-Value
PLR-2	>44	42 (84%)	18 (18%)	<0.001	23.92 (9.61–59.54)	<0.001
SII-2	>120	48 (96%)	35 (35%)	<0.001	44.57 (10.22–194.43)	<0.001
SIRI-2	>1.65	38 (76%)	51 (51%)	0.006	3.04 (1.43–6.49)	0.004
PIV-2	>190	45 (90%)	29 (29%)	<0.001	22.03 (7.95–61.10)	<0.001
HALP-2	≤9	43 (86%)	30 (30%)	<0.001	14.33 (5.79–35.47)	<0.001

MH = Mantel–Haenszel common odds ratio estimate *p* value, SIRI (systemic inflammatory response index), PLR (platelet/lymphocyte ratio), PIV (pan-inflammatory index), HALP (hemoglobin, albumin, lymphocyte, platelet index), and SII (systemic immune-inflammation index).

## Data Availability

The original contributions presented in this study are included in the article. Further inquiries can be directed to the corresponding author.
